# Comparison of the adaptive implementation and evaluation of the Meeting Centers Support Program for people with dementia and their family carers in Europe; study protocol of the MEETINGDEM project

**DOI:** 10.1186/s12877-017-0472-x

**Published:** 2017-04-04

**Authors:** R. M. Dröes, F. J. M. Meiland, S. Evans, D. Brooker, E. Farina, D. Szcześniak, L. D. Van Mierlo, M. Orrell, J. Rymaszewska, R. Chattat

**Affiliations:** 1 Department of Psychiatry, VU University medical center/GGZinGeest, Postbox 74077, 1070 BB Amsterdam, The Netherlands; 2 Association for Dementia Studies, University of Worcester, Henwick Grove, Worcester, WR26AJ UK; 3 Santa Maria Nascente IRCCS Clinical Research Center, Don Carlo Gnocchi Foundation, Via Alfonso Capecelatro 66, 20148 Milan, Italy; 4 Department of Psychiatry, Wroclaw Medical University, Pasteura 10, 50-367 Wroclaw, Poland; 5 Institute of Mental Health, University of Nottingham, Triumph Road, Nottingham, NG7 2TU UK; 6 Department of Psychology, University of Bologna, Viale Berti Pichat 5, Bologna, Italy

**Keywords:** Dementia, Carer competence, Meeting Centers Support Program, Person-centered approach, Implementation process, Effect evaluation

## Abstract

**Background:**

The MEETINGDEM study aims to implement and evaluate an innovative, inclusive, approach to supporting community dwelling people with mild to moderate dementia and their family carers, called the Meeting Centers Support Program (MCSP), in three countries in the European Union (EU): Italy, Poland and United Kingdom. Demonstrated benefits of this person-centered approach, developed in The Netherlands, include high user satisfaction, reduced behavioral and mood problems, delayed admission to residential care, lower levels of caregiving-related stress, higher carer competence, and improved collaboration between care and welfare organizations.

**Methods:**

The project will be carried out over a 36 month period. Project partners in the three countries will utilize, and adapt, strategies and tools developed in the Netherlands. In Phase One (month 1-18) activities will focus on establishing an initiative group of relevant organizations and user representatives in each country, exploring pathways to care and potential facilitators and barriers to implementing the program, and developing country specific implementation plans and materials. In Phase Two (month 19‑36) training will be provided to organizations and staff, after which the meeting centers will be established and evaluated for impact on behavior, mood and quality of life of people with dementia and carers, cost-effectiveness, changes in service use, user satisfaction and implementation process.

**Discussion:**

An overall evaluation will draw together findings from the three countries to develop recommendations for successful implementation of MCSP across the EU. If the Meeting Centers approach can be widely implemented, this could lead to major improvements in dementia care across Europe and beyond.

**Trial registration:**

The trial was retrospectively registered in May 2016: trial number: NTR5936.

## Background

### Background and state of the art

Dementia has substantial consequences for both persons with dementia and their families. Various support services and psychosocial interventions, e.g. home care, psychogeriatric day care, occupational therapy, psychoeducation and support groups for carers, are available to support them [1, 2]. However, people with dementia and their carers often delay asking for help as they may be afraid to become dependent on others or fear nursing home admission [3]. In addition, the available services are often fragmented, making it difficult for people to find the services that fulfil their individual needs and preferences. As a result many people have unmet needs [4]. This risks overburdening of informal carers and ‘accelerated’ nursing home admission.

Several systematic reviews demonstrated that multicomponent support programs, including combinations of information, practical, emotional and social support, attuned to the individual needs, are more effective than single support activities for people living with dementia or family carers (e.g. participation in day care or a support group) [5–7]. Overall it is concluded that the general mental health of people with dementia and carers are improved by combined support programs and that admission to long-term care is delayed [7]. Examples of positively evaluated combined programs include case management in combination with psychoeducation, skills training and behavior management for the caregiver [8], a multimodal 4-week treatment program to help people accept and adapt to the consequences of dementia [9], an environmental skill building program [10], occupational therapy at home [11], and the multicomponent Meeting Centers Support Program [12–15].

Although the added value of combined support programs has been demonstrated in scientific studies, their implementation remains limited in care practices across Europe. Research shows that dissemination and implementation of care innovations is not easy and certainly not a guaranteed consequence of proven effectiveness of these innovations. Implementation research that yields knowledge on these context related facilitators and barriers of implementation, as well as effective adaptive implementation strategies and materials (incl. the necessary training of organizations and staff), is therefore extremely important in order to advance the implementation of evidence based care innovations [16–19].

This study focuses on the further dissemination, implementation and evaluation of the Meeting Centers Support Program, which was evaluated positively and successfully disseminated in the Netherlands, to other European countries.

### The meeting centers support program

The predicted increase in the number and proportion of older people with dementia over the next 40 years highlights the need to identify ways to promote timely cost-effective interventions that help people with dementia to continue to live independently in the community as long as possible. In order to address this issue and solve the above mentioned problems that tend to delay timely care and support, the Meeting Centers Support Program (MCSP) offers an integrated package of care and support both for the person with dementia and for their informal carer(s), existing of, among other things, a social club for the person with dementia and psychoeducational meetings and discussion groups for the carers, and social activities and a weekly consultation hour for both as well as regular ‘center meetings’ for all involved in the program (see for a more detailed description of MCSP the Methods section).

The MCSP is based on the theoretical framework of the Adaptation-Coping model [12, 20].

This framework assumes that people with dementia and informal caregivers have to deal with adaptive tasks, such as coping with disabilities, changes in behavior and mood of the person with dementia and maintaining a positive self-image. The ability of people with dementia and informal caregivers to adequately cope with these tasks has an influence on their perceived burden as well as on mental and physical health problems [21].

The MCSP is person-centered, i.e. attuned to individual needs, abilities and wishes, and focuses on helping people deal with the changes dementia brings in their life, and supports them in living well with dementia. The program is offered in accessible locations that facilitate social inclusiveness and community integration. MCSP operates on the borders of social care, welfare and health, aiming to counteract the fragmentation of services that people and their families need at this stage. The efficacy of MCSP was demonstrated both for people with dementia and their carers, in two controlled multicenter studies in the Netherlands (1994‑1996 and 2000‑2003) in which MCSP was compared with traditional psychogeriatric day care in nursing homes in which carers were not offered a specific support program [13–15]. In both studies, compared to those using traditional day care, after 7 months of participation in the MCSP participants with dementia showed less behavioral and mood problems (less inactivity, unsocial and depressed behavior, and a higher self esteem) and nursing home admission was delayed (after 7 months 4% of the MCSP-participants were admitted to a nursing home compared to 30% of day care participants). Carers taking part in the MCSP generally felt more competent and less burdened than carers using day care as respite only [13], and lonely carers also reported fewer psychosomatic complaints. Patients and carers reported high levels of satisfaction with MCSP and the majority of carers felt supported by other carers [15].

Implementation research in the Netherlands identified various factors that promoted successful implementation of MCSP, including specific characteristics of the program (filling gaps in the care offer for the target group), experienced staff, adequate funding and good cooperation between care and welfare organizations [16, 17]. An implementation guide [22], film and training course for staff were prepared to help care and welfare organizations set up meeting centers, while a helpdesk supported dissemination of the MCSP approach. As a result the centers have spread across the country and today there are 144 centers in the Netherlands. Together they offer support to 3900 people with dementia and 3900 carers annually.

#### Aim of the study and research questions

The overall aim of this proposed implementation study is to prepare, support and evaluate the dissemination and implementation of the successful multicomponent Meeting Centers Support Program for people with dementia and their carers in three countries in Europe, (Italy, Poland and the United Kingdom).

The main research questions are:

1. Which care and welfare, and other, organizations in the countries participating in this implementation study, should be involved to successfully guide the implementation process within these countries?
2. What are the conditions for successful implementation of the Meeting Centers Support Program in these different European countries? In other words: What country specific facilitators and barriers are foreseen on beforehand and what facilitators and barriers are identified during the implementation process in the participating countries?
3. What country specific implementation plan, including implementation strategies and materials (toolkit), funding sources, is needed for adaptive implementation of the MCSP in the participating countries?
4. When implemented, are the results of the MCSP ((cost)effectiveness, user satisfaction) comparable with those found in the Netherlands?
5. How can the findings of the implementation study be disseminated to stimulate further dissemination and implementation of the MCSP in the participating countries and other countries in Europe?

## Methods/Design

### Design, participants and setting

A 3 year, two phase, implementation study will be conducted to a) explore and prepare and b) adaptively implement, evaluate and disseminate the Meeting Centers Support Program, as developed in the Netherlands, for people with mild to moderate dementia in three countries in Europe, i.e. Italy, Poland and United Kingdom. The reason why the MCSP will be implemented in these particular countries is because these countries had applied within the INTERDEM network to collaborate on a grant proposal for this implementation study within the EU Joint Program - Neurodegenerative Disease Research (JPND) project.

Within each participating country a national project team of at least one research institute will conduct the implementation study.

#### Phase 1: exploration and preparation

To answer the *research question 1* the project team in each country (except for the Netherlands) first will set up collaboration with a regional welfare and/or a care organization to initiate the project. In addition, collaboration will be sought with the national or regional Alzheimer organization in these countries as advocates of people with dementia and carers. To prepare and guide the practical implementation of the MCSP, in at least one region in each of the participating countries a projectleader, trained in the vision of the MCSP, and a multidisciplinary initiative group will be recruited comprising representatives of care and welfare, and if appropriate other organizations who are interested and willing to participate in the implementation project. This strategy using a community of practice approach to build a local initiative group to prepare and guide the implementation of a meeting center has proven to be a very effective implementation strategy in the Netherlands. With the help of this initiative group the structure and organization of the health care system relevant for dementia care in the participating countries will be explored and analyzed and the pathways to (timely) professional care for the person and their carer in each country will be identified and compared between countries.

Subsequently, to answer the first part of *research question 2* (foreseen facilitators and barriers), a survey will be conducted among care and welfare organizations to identify expected facilitators and barriers of implementation of the MCSP on a micro (primary process), meso (organizations) and macro level (law and regulations). The second part of research question 2 (facilitators and barriers during the implementation) will be investigated from the end of the preparation phase (retrospectively) until 1 year after implementation of MCSP (execution and continuation phase) in at least one region of each of the three partner countries. A survey will be conducted on facilitators and barriers at micro, meso and macro level.

To answer *research question 3*, based on the results of this survey an implementation plan with implementation strategies and materials (toolkit) will be developed for each country, utilizing and adapting already existing successful implementation strategies and materials developed in the Netherlands. In addition, a ‘train the trainer’ course will be developed.

#### Phase 2: implementation, evaluation and dissemination

In the implementation and evaluation phase, the national ‘train the trainer’ course will be offered in each of the participating countries to inform/train representatives of care and welfare organizations who are interested in implementing the MCSP in their own region. The MCSP will subsequently be implemented in at least one region in each participating country (Milan, Wroclaw, Worcestershire). The implementation will be conducted according to the country-specific implementation plans developed during the preparation phase and guided by an advisory board.

To answer *research question 4* the implementation of the MCSP will be evaluated (in each country and overall) regarding:

- (cost)effectiveness of the MCSP on behavior, mood, social support, experienced stigma and quality of life of people with dementia and loneliness, general health and sense of competence of their carers and delay of institutionalization, by means of a controlled trial (pre-test and post-test control group design) with measurements at Month 0 and Month 7, in which the MCSP will be compared with usual care in each country. This timing of measurements was chosen in line with the design of the previous research into the MCSP in the Netherlands to make comparison possible. The aim is to recruit people with mild to moderately severe dementia (Global Deterioration Scale score 4 and 5; mild to moderate dementia) and their carers, as these are the target group of the MCSP. There will be no restrictions on the age of people with dementia or carers participating in the program. In total 75 patient-carer dyads who will be offered the MCSP and 75 patient-carer dyads usual care. This number of dyads is based on a power analysis: in case of moderate effects (d = 0,5), a power of 0.80 and alpha 0.05, at least 64 dyads are needed. Taking into account a drop-out of 15% 75 dyads need to be recruited (25 per country). To check for comparability of the MCSP group and the control group (per country and overall), in each country at baseline the groups will be compared on the following characteristics: severity of the dementia and degree of assistance/care needed by the patient, and the sense of competence of the caregiver. During the intervention period possible longitudinal changes in the person with dementia will be monitored (illness, physical disability, medication and the use of other types of support than offered in the experimental or the control group). Reasons for drop out, and special (life) events in the period of 1 month before the measurements will also be checked. To compare the costs of the two conditions the following costs will be taken into account: service use, use of psychotropic medication, hospital admission and admission into a long term care setting (incl. temporary admission as respite for carer);
- user satisfaction among people with dementia and carers participating in the program by means of a survey and focus groups;

To answer *research question 5*, national and international dissemination plans will be developed.

The Medical Ethics Committee of the VU University medical center approved the study as non-medical scientific research.

### Adaptive implementation of the meeting centers support program

#### The intervention: the Meeting Centers Support Program

The Meeting Centers Support Program (MCSP) offers an integrated package of care and support:

- both for the person with dementia and for their informal carer(s). For the person with dementia a social club is organized (3 days per week), where they can participate in recreational activities and psychomotor therapy. For carers there are psychoeducational meetings and discussion groups. For both there are social activities, a weekly consultation hour and regular ‘center meetings’ that allow all participants, staff and volunteers to share experiences. The staff also helps to co-ordinate care services at home.
- attuned to individual needs. The support strategy is innovative in that it is fully attuned to the individual needs of participants. The MCSP is theoretically based on the Adaptation-Coping model [20] which explains how persons and carers adapt to, and cope with, the changes they experience in their lives because of the dementia, and how biological, psychological and social factors can influence this process. Based on the problems they may experience with adapting (e.g. with adapting to disabilities and maintaining an emotional balance, a positive self image and social relationships), an individual care and support plan is set up, which is evaluated regularly and adapted if necessary. Depending on the identified problems/needs/wishes, the support strategies for people with dementia vary from giving information to help them better deal with the changes in their lives, to trying to re-activate, re-socialize and optimize their emotional functioning [12]. Support strategies for family carers vary from giving information to offering practical, emotional and social support [13].
- that have been demonstrated to be beneficial for people with dementia and carers. The program integrates several support activities that have been shown to be effective for persons with dementia or their carers in research and/or practice. These include cognitive stimulation, activity groups, music therapy, psychomotor therapy, family support groups, psychoeducation and counselling.
- in an accessible location that facilitates social inclusiveness and community integration. The Meeting Centers are integrated in easy to access community centers, maximizing social integration with people from the local community and promoting social participation. This makes them more attractive than institutional day care (common in many EU countries) and makes it easier for people to use support from an early stage of the disease. Examples of activities that have developed in the centers spontaneously include: playing billiards and having a drink with visitors at the coffee bar, painting together, and interacting with other generational groups using the same community facilities. In addition, family carers participate in activities in the community center. Through contacts that users develop in the centers local solidarity is stimulated, volunteers for MCSP are easily recruited, and public attitudes towards dementia are positively influenced.
- On the border of care and welfare. To counteract the fragmentation of care and welfare services, the MCSP is offered by a small professional team and volunteers in close cooperation with other (multidisciplinary) professionals/organizations in the region that offer dementia care. These include general practitioners, memory clinics, home care agencies, mental health care organizations and nursing homes. Some of them participate in the delivery of the program by e.g. leading discussion groups or delivering informative lectures. This collaboration is formalized in a written agreement.

#### Methodology of adaptive implementation of MCSP

- Preparation of implementation: the initiative group
  The local initiative group in each country will prepare the implementation of the meeting center by developing a country specific implementation plan (see Research methods and measuring instruments: country specific implementation plans)
- Guidance of the implementation process
  In each region/site in each country, a trained project leader (pioneer/consultant) will guide the overall implementation process, with the aim to help the staff during all stages of building context, training and the actual implementation process. This trained consultant will also advise care and welfare services in other regions of the country that are interested in establishing the MCSP in their own region.
- Building the context and staff training
  Based on the implementation plan developed, in each country (region) a process of ‘building the context’ will be undertaken as preparation of the actual implementation of the MCSP. The main action will be to select staff/setting with high interest in developing the MCSP. The best type of organization capable of implementing the MSCP at the local level will be the pioneer organization; they will appoint the staff. After selection of the staff they will be informed on the country specific implementation plan and advised on how to set up the MSCP, including preparing and decorating the location, recruiting participants, collaborating with the local network of available welfare and care services, considering local law and norms regarding care, and implementing dissemination strategies in order to keep contact and interaction with the network on a micro and meso level. Furthermore, the staff will receive a course with detailed information on the MCSP and training in person-centered activities and psychosocial interventions for persons with dementia and carers. Topics will include the experience of living with dementia, the experience of being a carer, the vision and model of the MCSP (including access criteria and influence of persons with dementia and carers in shaping the program), available welfare and support services in the region, person-centered psychosocial care based on the adaptation-coping model, emotional burden for professional caregivers, theory and practice of psychomotor therapy. The training will be offered in an interactive way in order to motivate and encourage staff to participate and to ensure an adaptive implementation, taking into account not only the local context but also staff’s education levels and competencies.
- Starting the MCSP in practice
  After building the context and staff competence, the actual adaptive implementation of the MCSP will be started in at least one region/location per country (Milan, Wroclaw, Worcestershire) by the trained staff, guided by the trained pioneer/consultant and based on the prepared implementation plan developed by the initiative group. The first step of implementation is preparing and decorating an appropriate site/location according to the requirements described in the implementation plan and to arrange economic resources to cover the costs of the MCSP. These activities will commence already during the preparation phase and training process and will be continued (especially regarding arrangements for structural funding) throughout the implementation phase.
- The advisory group after starting the center
  For each region/location an advisory group of representatives of the care and welfare organizations that agreed to collaborate with the MCSP will be set up, and will guide the implementation process in the first year after starting the meeting center. The advisory group will meet at least every 3 months. Members of the initiative group who prepared the implementation plan will be invited to take part of this advisory group. To ensure user involvement in the implementation process the national Alzheimer organization will be invited as advocate of people with dementia and carers. The voice of people with dementia and the family carers participating in MCSP will be heard in the monthly center meetings.
- Funding of the MCSP
  The funding for a local meeting center (personnel: program coordinator, activity therapist, and/or care assistant and volunteers + material costs) needs to be arranged locally by each site. However, in each country the local organization who sets up the meeting center will be provided with starting-up funding (*this was covered by the research project*) for preparation (education of personnel and volunteers) and implementation costs (location decoration, publicity, advisory board meetings). The experience in the Netherlands is that this helps to stimulate care and/or welfare organizations to be involved in starting up the implementation of the MCSP and can help to stimulate other funding organizations to provide additional financial support. In order to arrange structural funding, contacts within the community and health insurance and charity sectors will be consulted.
- Recruitment of participants
  When the location and funding to start the MCSP are in place to cover at least 1 year of operations, the next step will be taken, i.e. the recruitment of participants, taking into account the access criteria set by each country/site. Although the core group of participants in each country will be persons with mild to moderately severe dementia and their caregivers, site specific access criteria can be defined for each country. Activities will be planned attuned to the participant’s needs, wishes and conditions, taken into account the adaptation areas (cognitive, emotional, social) in which they experience difficulties. A weekly staff meeting will be held to evaluate how activities are experienced by the participants, to plan adapted activities, and to discuss about new participants. Every 2 months a center meeting will be arranged with all participants (people with dementia, carers), staff and volunteers to discuss the experiences with the program and to get feedback from the participants to attune the MCSP as much as possible to their needs and wishes and to stimulate their feeling of having influence on the content of the program and the joint responsibility for ‘their club’, the meeting center.
- Pioneer workshops for further implementation and dissemination.
  To stimulate further implementation/dissemination of MCSP in the involved countries, a ‘train the trainer course’/pioneer workshop will be developed, and offered in each country. The aim of this implementation training is to inform and train representatives of care and welfare organizations in the participating countries, who are interested to implement the MCSP in their own region, how to implement the program. The course will provide information on the MCSP, its’ effectiveness as studied in the Netherlands, and information on how to implement MCSP based on the implementation plans prepared for each country and experiences from the national implementation of the MCSP in the Netherlands.

### Research methods, measuring instruments and procedures

#### Organizations to guide the implementation process (question 1)

First we will explore which care, welfare and other organizations involved in dementia care in the participating countries should be engaged in the implementation process of the Meeting Centers Support Program. The project team in each country, consisting of the academic partner and other partner research organization(s), a care organization and a welfare organization and a representative of the national or regional Alzheimer association, will 1) analyze the relevant care, welfare and other organizations and associations in the region/country; and 2) compile a list of potential partner organizations interested in the implementation of the MCSP, based on discussions with their representatives. All these organizations will be invited to an information meeting at which the MCSP and the aims and methodology of the implementation project are explained and people are invited to join the initiative group to set up a meeting center.

#### Identifying facilitators and barriers of implementation (question 2)

In previous research on successful implementation of MCSP in the Netherlands [22], a theoretical model for adaptive implementation was developed to inventory facilitators and barriers of implementation of MCSP [16, 17]. This model describes influencing factors on different levels of implementation, e.g. on a micro (primary process), meso (cooperation between organizations) and macro level (laws and regulations), and during different phases of the implementation process (preparation, execution, continuation).

Based on the results of this study, a questionnaire will be composed on potential facilitators and barriers, and translated in languages of the three countries. This questionnaire will also record characteristics of the innovation itself, i.e. the Meeting Centers Support Program, and other preconditions that can have a facilitating or impeding influence during the implementation process (preparation phase, execution phase, continuation phase) will be included.

This questionnaire will be discussed with organizations that agreed to take part in the initiative group. They will be asked to indicate which facilitators and barriers they foresee to influence the implementation of MCSP in their own country, as well as to describe additional expected facilitators and barriers that are not included in the checklist yet. The results will be discussed in the initiative groups, summarized per country and disseminated to all participating countries. Subsequently, the list of foreseen facilitators and barriers will be used by each initiative group to define promising implementation strategies. In each country the results will be summarized for each phase and level of implementation. This document will be sent to all participating countries and will inform the development of a detailed implementation plan in each country.

During the implementation phase a process analysis will be conducted for which quantitative and qualitative research methods will be used. In each participating country data will be collected from key figures in the implementation process, who are selected by means of ‘purposive sampling’ [23]. The following criteria will be used: representatives from different organizations (care and welfare) involved in the implementation of the MCSP, key figures with professional and financial expertise, and key figures at the local level (municipality) and at the regional/national level (e.g. Alzheimer association). Different types of key figures will be asked a selection of questions, depending on their area of expertise and involvement in the implementation process. Data will also be drawn from the minutes and reports of the initiative groups in the different countries to inform an inventory of facilitators and barriers to implementation.

##### Country specific implementation plans (question 3)

Based on the results of the inventory of (potential) facilitators and barriers for implementation of the MCSP and the proposed implementation strategies in each country, a country specific implementation plan will be developed by each initiative group to guide a successful implementation of the MCSP. The initiative group will be led by one of the partner organizations (to decide per country/region who takes the lead: a research center, care or welfare organization or Alzheimer association). The process to create the implementation plan will consist of several steps/tasks that need to be completed before the actual implementation can take place. This process takes approximately 1 year. The steps/tasks will include: definition of the target population (inclusion and exclusion criteria), content of the support program, requirements of the location and listing of locations that meet these requirements, cultural issues that are relevant to consider for the implementation (such as what is the cultural norm for dementia care?), number and educational background of personnel and required training, funding of the MCSP, collaboration with other organizations, and development of a communication plan in order to communicate the existence of the MCSP as a resource of support for persons with dementia and carers to appropriate stakeholders (e.g. health care professionals, volunteer associations for dementia, local social care workers, responsible local government, etc.). The initiative groups in each country will form subgroups to elaborate on the various steps/tasks in the action plan. The progress made in the subgroups will be discussed within the monthly plenary initiative group meetings.

Also, implementation materials will be developed, taking into account the regional context, that can support the provision of information about the MCSP among various target populations and create interest in the MCSP. Existing materials developed in the Netherlands will be adapted and translated as necessary. Implementation and dissemination materials that are already available in the Netherlands are: a DVD on the MCSP (also available in English), a flyer on the MCSP, a practical implementation guide [24], a course for personnel of the Meeting centers, scientific and professional publications (in English) on the effectiveness and implementation of the MCSP in the Netherlands, a website with information on the Meeting centers in the Netherlands, a Dutch LinkedIn group for people interested in or working in meeting centers.

#### Evaluation of (cost)effectiveness (question 4a)

To determine the severity of the dementia the Reisberg’s Global Deterioration Scale [25] will be used. Background information will be collected for all participants, including socio-economic status, gender and comorbidities, as well as who referred them to the MCSP/usual care, in order to identify the characteristics of the (groups of) people with dementia and carers who use the MCSP and the usual care and to learn more about the pathways to care in the different countries.

The selection of the outcome measures was based on the Adaptation-Coping model (cognitive, emotional and social adaptation) [20] and previous research into the effectiveness of MCSP on behavior, mood and quality of life of people with dementia and general health, caregiver distress, sense of competence of the carers [12, 13].

For the person with dementia the NeuroPsychiatric Inventory (NPI) [26], the Cornell scale for depression in dementia [27], Duke social support inventory [28], Stigma Impact Scale: Neurological Impairment [29], the subscale self-esteem of the Dementia Quality of Life questionnaire (DQOL) [30], and the Quality of Life-Alzheimer’s Disease Scale (QOL-AD [31] will be used. For the carers the UCLA Loneliness scale [32], General Health Questionnaire-12 [33], NPI-caregiver distress scale [26], and the Short Sense of Competence Scale (SSCQ) [34] will be used as an outcome measure. Data collection on all outcome measures will occur at baseline and after 6 months of support.

For evaluation of the cost-effectiveness, data will be collected on: service use (incl meeting center services used by person with dementia and carer), psychotropic medication, admissions into a hospital and into a long term care setting (incl. temporary admission as respite for the carer), carer time spent on caring. For this data collection specific questionnaires will be administered from the carers at Month 0 and 7. In addition, cost diaries are filled in by the carers during the whole intervention period.

#### User evaluation (question 4b)

In order to assess the opinions of the people with dementia and their carers on the support they receive, and on the various elements in the support program, all new participants in the support program who agree to take part in the study will be interviewed in-depth, after 3 and 6 months of participation. The interview will utilize a questionnaire about the different program elements. The people with dementia will be interviewed by an independent interviewer, the carer will receive a written questionnaire and will be asked to complete it and to send it directly to the research team. The two questionnaires that were developed for this user evaluation among people with dementia and their carers in the Netherlands [12] will be translated in the native languages of the participating countries.

A qualitative evaluation will also be undertaken in each meeting center by means of separate focus groups for people with dementia and carers (a total of 2 focus groups per MCSP) to explore their experiences with the MCSP. The focus groups will be recorded and transcribed.

#### Dissemination strategies (question 5)

In collaboration with the care and welfare organizations, Alzheimer organizations, members of the initiative groups and other relevant stakeholders in the participating countries, the project team in each country will develop a country specific plan to disseminate the project results and stimulate further dissemination and implementation of MCSP in these countries. An international dissemination plan will also be developed, including a project website to raise (inter)national awareness of the proposed project and to disseminate the emerging findings, publications in scientific and professional journals, the newsletters of the national Alzheimer organizations and Alzheimer Europe, and presentations on conferences for scientists, professionals and the general public. In addition a practical implementation guide and toolkit will be developed.

During the project period a network of interest will be established by creating and updating a database with contact details of stakeholders who have interest in the MCSP in the participating and other EU countries. This network will be informed on the progress of the project by means of an e-newsletter, including details of the training courses and the developed implementation materials.

At the end of the project, in each country a final national/regional event will be organized to present the findings of the implementation study to relevant stakeholders, such as (in)formal carers, care and welfare organizations, policy makers, local government, health insurers and charity.

### Overall project structure and time plan

**Fig. 1:**
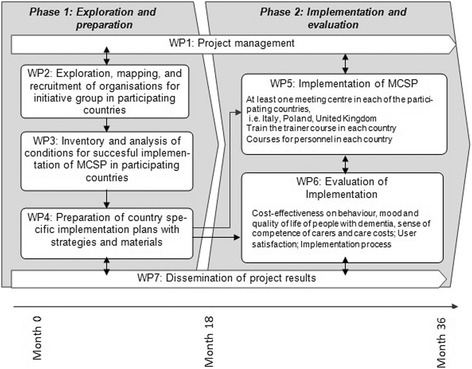
Structure of MEETINGDEM project: Workpackages and interrelationships

The two project phases (1: Exploration and preparation; 2: Implementation and evaluation) will be carried out in 18 months each (total 36 month). The work is divided into seven workpackages (WPs; see Fig. 1):

1. *Project management (Month 1‑36):* Internal and external project management, ensuring that the project’s main objectives are realized on schedule and according to budget.
2. *Exploration, mapping and recruitment of organisations (Month 1‑3):* care and welfare organisations providing dementia care in each country will be invited for the initiative group; this initiative group will provide the information needed in WP 3, 4 and 5.
3. *Identification of conditions for implementation of MCSP in participating countries (Month 3‑6):* Based on the results of the Dutch implementation study [16] *foreseen* facilitators and barriers will be inventoried by the initiative groups in each country. Promising solutions and implementation strategies will be described. This will be input for WP 4.
4. *Preparation of implementation strategies and materials (Month 7‑17):* The country specific implementation plans will be prepared. Already existing Dutch implementation strategies and materials will be used and adapted, if necessary. This will be input for WP 5.
5. *Implementation of MCSP (Month 16‑36):* A course is developed and offered to the personnel of the meeting centers. In addition, the pioneer workshops are provided for organizations who are interested to set up a meeting center in their own region. In each participating country the MCSP is adaptively implemented in at least one region, in one meeting center.
6. *Evaluation of implementation (Month 19‑36):* The efficacy and cost-effectiveness of the implementation of the MCSP, the satisfaction of the participants (people with dementia and carers) and the implementation process will be evaluated in at least one region per country.
7. *Dissemination of project results (Month 1‑36):* The results on implementation of the MCSP will be disseminated in the participating countries, in Europe, and worldwide, based on (inter)national dissemination plans. A database on interested stakeholders will be created to form the network of interest.

### Analysis

#### Process evaluation (question 2)

The data on facilitators and barriers of implementation, collected in the different implementation phases by the developed checklist, will be analyzed descriptively for each phase and level (micro, meso, macro), and for each country and overall (international). In addition, differences between countries will be analyzed. Finally, a comparison will be made with the results from the previous study into the implementation of MCSP in the Netherlands.

The written material obtained from the minutes and reports of the initiative groups meetings in the different countries will be analyzed qualitatively on mentioned facilitators and barriers of implementation during the preparation phase. The material will be read and coded by two independent researchers who will assign key words to extracts of the text. Codes will be assigned based on the checklist of possible facilitating and impeding factors (deductive method). New codes will be added if necessary (inductive method). In case of disagreement, the assessors will discuss the coding in detail until consensus is reached. After that all coded texts will be arranged by theme and summarized for each country and compared with each other. The main similarities and differences between the countries will be summarized. To aid the qualitative analysis a computer program will be used.

#### Evaluation of effectiveness and cost-effectiveness (question 4a)

The baseline characteristics of participants in the MCSP group and control group will be analyzed descriptively and differences between the groups will be tested (two-sided, alpha 0.05) by t-tests and Chi2 tests, depending on the type of data (ordinal, nominal). The data collected on the outcome measures will be analyzed by covariance analyses (ANCOVA’s) on the posttest measurements including the baseline measurements as covariates in the analysis. In addition, potential confounding variables will be included as covariates, i.e. characteristics that differ between the groups at baseline and are correlated with one or more outcome measures. For each outcome measure the effect size will be calculated according to Cohen (1977; small effect d = 0.2; moderate effect d = 0.5; large effect d > 0.8).

The cost-effectiveness analysis will compare the outcomes and costs of the MCSP at the 7 month follow-up (a) from a health and social care perspective, and (b) from a societal perspective (including unpaid carer costs). Cost-effectiveness outcome measure will be: Incremental cost per quality-adjusted life year (QALY), using self-reported EuroQol EQ-5D-5 L [35].

#### User evaluation (question 4b)

The data collected with questionnaires will be analyzed descriptively. The transcribed texts of the recorded focus groups will be analyzed based on the principles of grounded theory (themes will be identified and text fragments will be coded by two independent researchers; discrepancies will be discussed until agreement is reached; finally all texts for each code will be summarized). In the reports on the user evaluation in each country anonymized citations will be used to explain the summarized results on themes mentioned by the people with dementia and the carers.

## Discussion

The MEETINGDEM project intends to adaptively implement and evaluate the Meeting Centers Support Program in three European countries. In Phase One activities will focus on establishing an initiative group of relevant organizations and user representatives in each country, exploring pathways to care and potential facilitators and barriers to implementing the program, and developing country and context-specific implementation plans and materials. In Phase Two training will be provided to organizations and staff, after which the meeting centers will be established and evaluated for impact, cost-effectiveness, user satisfaction and implementation process.

The study will provide relevant information on conditions for successful implementation of the combined MCSP for people with dementia and their carers in three very different European countries (Italy, Poland and the UK), which will be useful also for other countries in Europe who are interested to implement MCSP, or other combined support programs. It will also provide information on the effect of MCSP on behavior, mood, social support, experienced stigma and quality of life of people with dementia as well as loneliness, general health, caregiver distress and sense of competence of informal caregivers, and on the costs of MCSP in the different countries. This will enable us to compare the results of the MCSP in other European countries with those previously found in the Netherlands in terms of clinical outcomes and costs.

The project will deliver different type of data, results and products (‘deliverables’) of which some will be posted on the public project website (www.meetingdem.eu), such as: regional data bases of national/regional organizations involved in dementia care; initiative groups of representatives of organizations who prepare the adaptive implementation of MCSP in the participating countries; a report on the structure of the health care system and the patient and caregiver ‘pathway to a professional for dementia care’ in the three participating countries; a translated checklist (in English, Italian and Polish) on facilitators and barriers of implementation of MCSP; country specific implementation plans and materials (toolkit); a ‘train the trainer’/pioneer course; a course for personnel per country; trained personnel; operational meeting centers in each country; people with dementia and carers participating in the MCSP; a guide and toolkit for supporting successful implementation of the MCSP in Europe; scientific and professional publications and congress lectures on the efficacy, cost-effectiveness and user satisfaction of MCSP in different European countries, and on facilitators and barriers when implementing MCSP; (inter)national plans for dissemination of MCSP, a project website reporting on the implementation of MCSP in Europe; a database with stakeholders in Europe who are interested in the MCSP; and finally, in each country a national dissemination event.

Because the (cost)effectiveness study is conducted within an implementation study this may entail the following risks: the MCSP is not yet fully implemented during the experimental period or the personnel does not yet fully work according to the vision and principles of MCSP. This will decrease the effectiveness of the intervention, and may lead to recruitment problems in the starting period. If the first meeting centers in the three countries do not reach the number of participants required according to the power analysis (25 per country, taking into account an expected drop-out rate of 15%), more meeting centres are needed to be included in the study. This may be difficult due to funding and finding suitable locations. To minimize these risks a long preparation period of 12 months is planned, before the actual start of the centers. This will give the initiative groups enough time to prepare the implementation, arrange the necessary funding and location(s), communicate the new service offer to public and care and welfare providers in the region, recruit participants and train the personnel. Also during the first year of implementation, recruitment of participants will be continued (9 months) and, as a follow-up of the training, the personnel will receive supervision and guidance from one of the trainers and trained project leader in each country (five group supervision meetings).

Though country specific implementation plans will be created, the three meeting centres may encounter different barriers to implementation (such as difference in staff competencies and collaboration with other care partners), which can have an effect on the care received by people with dementia and their informal caregivers. A clear description of the adaptive implementation of MCSP in practice will help to identify these differences and if necessary, take these into account in the effect analyses.

There is a great need for high quality implementation research to demonstrate how care interventions can be put into practice in a variety of settings. Hence, this study of MCSP should make a major contribution to our understanding of the difficulties and key factors involved in making things work in other countries and services by providing an effective model for implementation which could be adapted for other care interventions. The results will also help in future policy and decision making on post diagnostic support and care for people with dementia and their carers. This will promote further dissemination of MCSP in Europe and may also serve as an example for dissemination and implementation of other effective psychosocial interventions for people with dementia and carers in and outside Europe.

## Abbreviations

MCSP: Meeting Centres Support Program
EU: European Union
JPND: EU joint programme- neurodegenerative disease research
NPI: Neuropsychiatric inventory
DQOL: Dementia quality of life questionnaire
QOL-AD: Quality of Life-Alzheimer’s Disease Scale
SSCQ: Short sense of competence questionnaire
WP: Workpackage
QALY: Quality-Adjusted Life Year

## References

[1] Moniz-Cook E, Manthorpe J (2008). (INTERDEM-network) Psychosocial interventions in early stage dementia; a European evidence-based text.

[2] Dröes RM, Van Mierlo LD, Meiland FJM, Van der Roest HG (2011). Memory problems in dementia: Adaptation and copingstrategies, and psychosocial treatments. Expert Rev Neurother.

[3] Van Mierlo LD, Meiland FJM, Dröes RM (2012). Dementelcoach: effect of telephone coaching on carers of community dwelling people with dementia. Int Psychogeriatr.

[4] van der Roest HG, Meiland FJ, Maroccini R, Comijs HC, Jonker C, Dröes RM (2007). Subjective needs of people with dementia: a review of the literature. Int Psychogeriatr.

[5] Acton GJ, Kang J (2001). Interventions to reduce the burden of caregiving for an adult with dementia: a meta analysis. Res nurs health.

[6] Brodaty H, Green A, Koschera A (2003). Meta-analysis of psychosocial interventions for caregivers of people with dementia. Am J Psychiatry.

[7] Smits CH, de Lange J, Dröes RM, Meiland FJM, Vernooij-Dassen M, Pot AM (2007). Effects of combined intervention programmes for people with dementia living at home and their caregivers: a systematic review. Int J Geriatri Psychiatry..

[8] Aupperle PM, Coyne AC (2000). Primary vs subspecialty care: a structured follow-up of dementia patients and their caregivers. Am J Geriatr Psychiatry.

[9] Romero B, Wenz M (2002). Concept and effectiveness of a treatment program for patients with dementia and their relatives. Results from the Bad Aibling Alzheimer Disease Therapy Center. Z Gerontol Geriatr.

[10] Gitlin LN, Winter L, Corcoran M, Dennis MP, Schinfeld S, Hauck WW (2003). Effects of the home environmental skill-building program on the caregiver-care recipient dyad: 6-month outcomes from the Philadelphia REACH Initiative. Gerontologist.

[11] Graff MJ, Vernooij-Dassen MJ, Thijssen M, Dekker J, Hoefnagels WH, OldeRikkert MG (2007). Effects of community occupational therapy on quality of life, mood, and health status in dementia patients and their caregivers: a randomized controlled trial. J Gerontol A Biol Sci Med Sci.

[12] Dröes RM, Breebaart E, Van Tilburg W, Mellenbergh GJ (2000). The effect of integrated family support versus day care only on behavior and mood of patients with dementia. Int Psychogeriatr.

[13] Dröes RM, Breebaart E, Meiland FJM, Van Tilburg W, Mellenbergh GJ (2004). Effect of Meeting Centres Support Programme on feeling of competence of family caregivers and delay of institutionalization of people with dementia. Aging Ment Health.

[14] Dröes RM, Meiland FJM, Schmitz M, Van Tilburg W (2004). Effect of combined support for people with dementia and carers versus regular day care on behaviour and mood of persons with dementia: results from a multi-centre implementation study. Int J Geriatri Psychiatry.

[15] Dröes RM, Meiland FJM, Schmitz M, Van Tilburg W (2011). An evaluation of the Meeting Centres Support Programme among persons with dementia and their carers. Nonpharmacol Therapies Dementia.

[16] Meiland FJM, Dröes RM, De Lange J, Vernooij-Dassen M. Development of a theoretical model for tracing facilitators and barriers in adaptive implementation of innovative practices in dementia care. Arch Gerontol Geriatr Suppl. 2004;(9):279–90. 10.1016/j.archger.2004.04.038 15207425

[17] Meiland FJM, Dröes RM, De Lange J, Vernooij-Dassen M (2005). Facilitators and barriers in the implementation of the meeting centres model for people with dementia and their carers. Health Policy.

[18] Grol R, Wensing M (2004). What drives change? Barriers to and incentives for achieving evidence-based practice. Med J Aust.

[19] Moniz-Cook E, Vernooij-Dassen M, Woods B, Orrell M, Interdem Network (2011). Psychosocial interventions in dementia care research: The INTERDEM manifesto. Aging Ment Health.

[20] Dröes RM (1991). In beweging: Over psychosociale hulpverlening aan demente ouderen. Academisch Proefschrift Vrije Universiteit Amsterdam.

[21] Dröes, RM, Lindeman, EM, Breebaart, E, van Tilburg, W Determinanten van belasting van verzorgers van mensen die lijden aan dementie. [Determinants of burden of carers of people who suffer from dementia]. In R. M. Dröes (ed.), Amsterdamse Ontmoetingscentra: een nieuwe vorm van ondersteuning voor dementerende mensen en hun verzorgers. Amsterdam: Vrije Universiteit, Afdeling Psychiatie; 1996. 89–118

[22] Dröes RM, Meiland FJM, De Lange J, Vernooij-Dassen M, Van Tilburg W (2003). The meeting centres support programme; an effective way of supporting people with dementia who live at home and their carers. Dementia.

[23] Barbour RS (1999). The case for combining qualitative and quantitative approaches in Health Services Research. J Health Serv Res Policy.

[24] Dröes RM, Van Ganzewinkel J. Draaiboek Ontmoetingscentra voor mensen met dementie en hun verzorgers. 3rd ed. Amsterdam; 2014.

[25] Reisberg B, Ferris S, de Leon MJ, Crook T (1982). The Global Deterioration Scale for Assessment of Primary Degenerative Dementia. Am J Psychiatry.

[26] Cummings JL, Mega M, Gray K, Rosenberg-Thompson S, Carusi DA Gornbein J (1994). The Neuropsychiatric Inventory: comprehensive assessment of psychopathology in dementia. Neurology.

[27] Alexopoulos GS, Abrams RC, Young RC, Shamoian CA (1988). Cornell Scale for Depression in Dementia. Biol Psychiatry.

[28] George LK, Blazer DG, Hughes DC, Fowler N (1989). Social support and the outcome of major depression. Br J Psychiatry.

[29] Burgener SC, Berger B (2008). Measuring perceived stigma in persons with progressive neurological disease Alzheimer's dementia and Parkinson's disease. Dementia.

[30] Brod M, Stewart AL, Sands L, Walton P (1999). Conceptualization and measurement of quality of life in dementia: The Dementia Quality of Life instrument (DQoL). Gerontologist.

[31] Logsdon RG, Gibbons LE, McCurry SM, Teri L (1999). Quality of life in Alzheimer's disease: Patient and caregiver reports. J Ment Health Aging.

[32] Hughes ME, Waite LJ, Hawkley LC, Cacioppo JT (2004). A Short Scale for Measuring Loneliness in Large Surveys. Results From Two Population-Based Studies. Res Aging.

[33] Goldberg D, Williams P (1988). A user’s guide to the General Health Questionnaire.

[34] Vernooij-Dassen MJ, Felling AJ, Brummelkamp E, Dauzenberg MG, van den Bos GA, Grol R (1999). Assessment of caregiver's competence in dealing with the burden of caregiving for a dementia patient: a Short Sense of Competence Questionnaire (SSCQ) suitable for clinical practice. J Am Geriatr Soc.

[35] Herdman M, Gudex C, Lloyd A, Janssen MF, Kind P, Parkin D, Bonsel G, Badia X (2011). Development and preliminary testing of the new five-level version of EQ-5D (EQ-5D-5L). Qual Life Res.

